# Combining Unguided Web-Based Attentional Bias Modification and Affective Working Memory Training to Decrease Anxiety: A Randomized Controlled Trial

**DOI:** 10.1007/s10608-024-10565-y

**Published:** 2025-01-09

**Authors:** M. D. Nuijs, H. Larsen, A. M. Klein, R. P. P. P. Grasman, R. W. Wiers, E. L. de Voogd, E. Salemink

**Affiliations:** 1 Developmental Psychology, Adapt Lab, Research Priority Area Yield, University of Amsterdam, Amsterdam, The Netherlands; 2 Psychological Methods, University of Amsterdam, Amsterdam, The Netherlands; 3 Department of Clinical Psychology, Clinical Psychology, Utrecht University, P.O. box 80140, Utrecht, The Netherlands; 4 Altrecht Academic Anxiety Center, Altrecht GGz, Utrecht, The Netherlands; 5 Developmental and Educational Psychology, Leiden University, Leiden, The Netherlands; 6 Institute of Urban Mental Health, University of Amsterdam, Amsterdam, The Netherlands

**Keywords:** Attentional bias modification, Working memory training, Web-based, Adults, Trait anxiety, State anxiety

## Abstract

**Purpose:**

Cognitive vulnerability to anxiety can partly be explained by an interplay of attentional biases and control processes. This suggests that when aiming to reduce anxiety, simultaneously reducing an attentional bias for threat and strengthening control processes would be the optimal approach. We investigated whether a combined web-based Attentional Bias Modification (ABM) with affective Working Memory Training (WMT) was effective in reducing trait anxiety relative to control conditions and whether state anxiety moderated ABM effects.

**Methods:**

In this pre-registered randomized controlled trial, adults with heightened trait anxiety (*n* = 433) received either an active or control visual search ABM combined with an active or control emotional chessboard WMT training (2 × 2 design). Trait anxiety (primary outcome) was assessed at pre- (T1), mid- (T2), and post-training (T3), and at 1, 2, and 3-months follow-up. Attentional Bias (AB) and Working Memory (WM) capacity were assessed at T1, T2, T3, and FU3. State anxiety was measured prior to each training session.

**Results:**

Irrespective of the training conditions, participants showed a decrease in trait anxiety over time. The ABM training was associated with stronger reductions in AB. The WMT training was not associated with more improvements in WM capacity relative to the control condition. No moderating effects of state anxiety, baseline AB or WM capacity were observed.

**Conclusions:**

The findings suggested that the current type of ABM combined with WMT in a web-based format, without therapist support, was not effective in reducing trait anxiety beyond control conditions.

The study was registered in the Netherlands Trial Register (NTR-NL4525, https://www.onderzoekmetmensen.nl/en/trial/23135).

**Supplementary Information:**

The online version contains supplementary material available at 10.1007/s10608-024-10565-y.

## Introduction

Anxiety disorders are the most common group of mental disorders with a 12-month prevalence of approximately 14% in Europe (Wittchen et al., 2011). The course of anxiety disorders is quite persistent (Bruce et al., 2005) and many individuals still experience symptoms after cognitive-behavioral therapy (Springer et al., 2018). Despite the burden of anxiety, many individuals fail to seek treatment (Olfson et al., 2000). Web-based interventions have the potential to break down barriers as they are easy to access, suitable for wide-scale implementation, and have low financial costs. Interventions that can be easily transferred to a web-based format are computerized cognitive trainings (Gober et al., 2021). The goals of this study were twofold: (1) to investigate whether web-based computerized attentional bias training and affective working memory training would be effective in reducing trait anxiety and (2) to investigate moderators of training effectiveness.

Cognitive models of anxiety propose several individual difference factors that jointly contribute to a cognitive vulnerability to anxiety, including strong threat-related associations and low levels of cognitive control or working memory capacity (Mathews & Macleod, 2005; Mogg & Bradley, 2016; Ouimet et al., 2009). It has been put forward that anxiety disorders are likely to arise when the strength of emotional activation exceeds the capacity for control (Mathews & Macleod, 2005). When threat-related and non-threat-related stimuli are present in the environment, there is enhanced orientation towards the threatening stimulus, increased attentional engagement and impaired attentional disengagement with the threatening stimulus in anxious individuals (Ouimet et al., 2009). This selective attention to threat could be overcome by high levels of cognitive control, such as working memory capacity. Thus, individual differences in two kinds of cognitive processes (attentional information processing biases and control processes) and their interplay are hypothesized to play a role in the development and maintenance of anxiety disorders (see also Mogg & Bradley, 2016). This may suggest that in order to reduce anxiety, interventions should simultaneously reduce selective attention to threat as well as increase the strength of control processes.

With respect to attentional processes, studies have demonstrated that individuals with elevated levels of anxiety are prone to selectively attend to threatening stimuli (Bar-Haim et al., 2007; Cisler et al., 2010) and theoretical models argue that this attentional bias (AB) for threat plays a role in the onset and maintenance of anxiety (Mathews & Macleod, 2005). Subsequent experimental studies manipulated AB and demonstrated effects on anxiety (for reviews see Macleod & Clarke, 2015; Van Bockstaele et al., 2014); providing evidence consistent with a causal role of AB in anxiety. Clinically interesting is the possibility to reduce ABes to threat with the aim of reducing anxiety. Attention Bias Modification (ABM) training is a computerized training in which participants practice allocating attention to neutral or positive stimuli over negative stimuli. While some meta-analyses reported non-significant effects from ABM on anxiety symptom reduction compared to control conditions (Cristea et al., 2015), others reported more promising, significant effects on anxiety (Fodor et al., 2020, when post-traumatic stress trials were excluded; Linetzky et al., 2015; Price et al., 2016 depending on specific conditions). Generally, there is heterogeneity in ABM’s effect on anxiety (see also Vrijsen et al., 2024) and there are large prediction intervals in relation to ABM’s effect on anxiety when compared with waitlist or sham training (Fodor et al., 2020), and it has been suggested that future studies should include larger sample sizes and examine relevant moderators.

One potential moderator might be context of training as meta-analyses suggested that lab-based ABM studies yielded larger effect sizes than web-based or home-based studies (Cristea et al., 2015; Fodor et al., 2020; Martinelli et al., 2022). A possible explanation for this difference is that participants likely feel more anxious and aroused in the lab compared to their own home. Given that moderate levels of arousal have been associated with better learning performances (Yerkes & Dodson, 1908), increased anxiety and arousal might enhance training effects (Nuijs et al., 2020). In line with this idea, Kuckertz et al. (2014) provided preliminary evidence that ABM combined with anxiety induction was more effective in reducing AB and social anxiety symptoms than ABM without a mood induction. Therefore, the aim of the current study was to examine the effectiveness of ABM training in changing anxiety and innovatively test whether naturally occurring increased levels of state anxiety prior to training sessions enhance ABM’s effects.

Whereas many studies investigated whether training AB can impact anxiety symptoms, less studies investigated whether improving control processes can impact mood and anxiety symptoms (Iacoviello & Charney, 2015). Working Memory (WM) functioning seems a promising target for anxiety-related interventions as in general, cognitive control deficits (Paulus, 2015) and specifically WM functioning impairments (Moran, 2016) have been observed in individuals with elevated levels of anxiety. As such, WM training might be a valuable intervention for anxiety by improving control processes and thereby possibly decreasing anxiety symptoms. However, initial findings from meta-analyses regarding WM training concluded that there was no convincing evidence for far transfer effects (thus beyond the trained process) when compared with an active control condition (Melby-Lervåg et al., 2016). More recently, the crucial distinction between WM in neutral versus affective contexts has been highlighted (Schweizer et al., 2019). That is, WM performance of individuals with mental health problems was specifically impaired by negative, affective material compared to neutral material; suggesting that specifically *affective* WM capacity plays a role in poor mental health. Relatedly, when directly comparing the effectiveness of affective versus neutral WM training (Minihan et al., 2021), findings suggested that affective WM training resulted in greater reductions in test anxiety than neutral WM training. In sum, the evidence regarding the effectiveness of neutral WM training is mixed, while findings with respect to affective WM training seems more promising in the context of anxiety.

The current study investigated the effectiveness of combining web-based ABM and affective WM training in decreasing trait anxiety and the potential role of state anxiety in ABM training effects. Adults with heightened trait anxiety were randomized to ten training sessions in which they received ABM (either active or control) and affective WM training (either active or control) in a 2 × 2 design. The active version of ABM consisted of a visual search paradigm where participants had to repeatedly find one positive emotional (i.e., happy) face in a 4 × 4 grid with 15 negative emotional faces. In the control version of ABM, participants had to find the only 5-petaled flower in a grid with 15 7-petaled flowers (de Voogd et al., 2014). Visual search training may offer a promising alternative to other ABM paradigms as the training instructions are more explicit concerning the valence of the stimuli (i.e., ‘to search for the positive face’) and hence may facilitate goal-directed processing (Mogg & Bradley, 2018). The active version of affective WM training consisted of an adaptive WM Training with Emotional Distractors (WMT-ED; De Voogd et al., 2016a) and the control version of a non-adaptive version of the same training. For unguided, web-based training, it is important to keep participants engaged with the training. Therefore, before the start of training, participants were asked to formulate personal anxiety-related goals that they aim to achieve by participating in the training and to formulate their most important reasons to change. In addition, they could earn points based on performance during training.

The pre-registered primary outcome was trait anxiety which was assessed at pre-, mid-, and post-training and follow-up at one, two, and three months. The cognitive outcome measures were AB and WM capacity which were assessed at pre-, mid- and post-training and 3 months follow-up. We hypothesized that the double active training condition (active ABM & active WMT-ED condition) would outperform the other conditions and that the single training conditions (active ABM & control WMT-ED; control ABM & active WMT-ED) would outperform the double control condition in improving trait anxiety (primary outcome) and the cognitive outcomes of AB and WM capacity (secondary outcomes). Secondly, we hypothesized that higher levels of state anxiety prior to training would result in stronger effects of ABM (pre-registered moderator) on trait anxiety, AB, and WM capacity. In addition, we exploratively examined whether higher AB and lower WM capacity at baseline would result in stronger training effects.

## Methods

### Design

This study was a 2 (ABM: active versus control) × 2 (WMT-ED: active versus control) factorial design with trait anxiety as the primary outcome, and cognitive changes (in AB and WM capacity) as well as moderation by state anxiety registered as the secondary outcomes.^1^ The study was approved by the Ethics Committee of Psychology department of the University of Amsterdam and was registered in the Netherlands Trial Register (NTR-NL4525, https://www.onderzoekmetmensen.nl/en/trial/23135).

### Participants

From June 2013 onwards, participants could sign up for an unguided, free, fully automated, and open access web-based training targeting anxiety. The inclusion criteria for this study were being aged 18 and older and a screening-score above 40 on the Dutch version of the State-Trait Anxiety Inventory Trait Scale (STAI-T) (Spielberger et al., 1983; Van der Ploeg et al., 1980). Participants who scored lower than 40 on the STAI-T during screening were allowed to participate in the training, but received a message that the training was possibly irrelevant for them; they were not included in the analyses. Based on Fisher and Durham (1999), a score of > 46 on the STAI-T was considered as having elevated trait anxiety levels and these participants were included in the study (and the analyses) and advised to contact their doctor for professional help.

Randomization was stratified by gender with a 1:1:1:1 ratio and a computerized procedure. Participants were blinded to their treatment allocation. The information letter stated that participants would receive two training paradigms and that each of them had two versions; a version which was expected to be effective and another version of which a smaller or no effect was expected. Participants were informed that for each training, there was a 50% chance to receive the ‘effective’ training and when interested, that participants would receive the effective versions of both training paradigms if they completed the training.

**Fig. 1:**
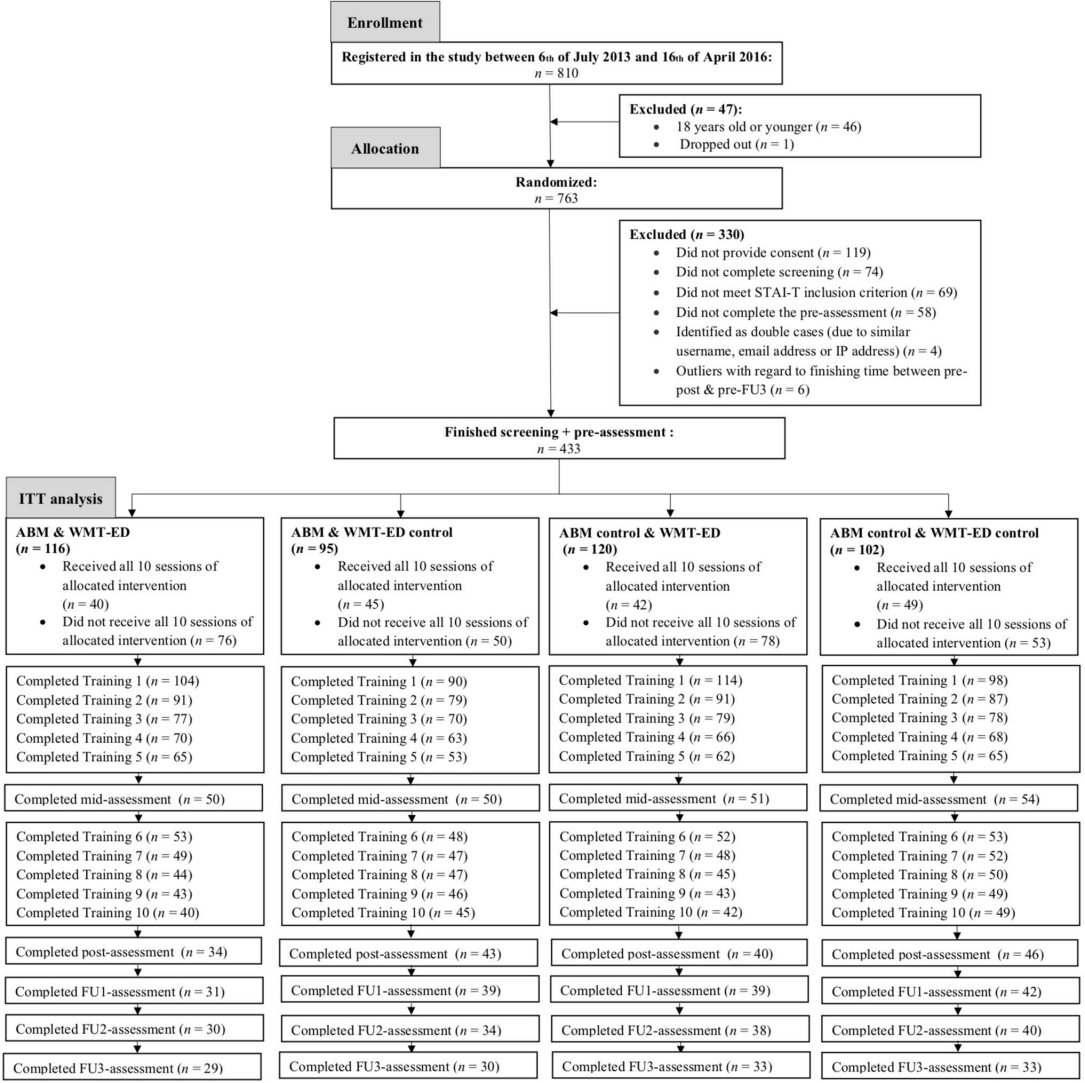
CONSORT Flow Diagram. *ABM* Attentional Bias Modification; *WMT-ED* Working Memory Training with Emotional Distractors; *IP address* Internet Protocol address; *ITT* intention-to-treat. A completed training session or assessment was defined as completing all elements of that session or assessment

Of the 810 participants who registered online to take part in the training, 763 were randomized over the 4 training conditions, see Fig. 1. Three hundred thirty participants were excluded from the analyses for various reasons, see Fig. 1. Six participants were excluded as the time between finishing the pre- to post-assessment (*n* = 3) or pre-assessment to FU3 (*n* = 3) was 2.5 *SDs* longer than the average time participants finished the assessments (the final model’s conclusions did not change when including these participants). The remaining participants (*n* = 433) were included in the intention-to-treat analyses (66.3% female; *M*age = 41.02; *SD* = 14.61; range = 18–84 years; 89.6% Dutch; 40.6% university educational level; 41.1% with high income; 44.3% used any type of medication; see Table 1). Of all participants who started the training, 47.3% (*n* = 205) completed T2, 40.6% (*n* = 176) completed all 10 training sessions, 37.6% (*n* = 163) completed T3, 34.9% (*n* = 151) completed FU1, 32.8% (*n* = 142) completed FU2, and 28.9% (*n* = 125) completed FU3.

**Table 1:** Demographic characteristics of the four training conditions

		ABM & WMT-ED		ABM & WMT-ED control		ABM control & WMT-ED		ABM control & WMT-ED control	
		(*n* = 116)		(*n* = 95)		(*n* = 120)		(*n* = 102)	
		Mean/n	SD/%	Mean/n	SD/%	Mean/n	SD/%	Mean/n	SD/%
Age, mean (*SD*)		40.45	14.61	41.45	13.62	41.28	15.62	40.96	14.44
Female, *n* (%)		68	58.6	69	72.6	78	65.0	72	70.6
*Nationality, n* (%)									
Dutch		100	86.2	88	92.6	106	88.3	94	92.2
Non-Dutch		16	13.8	7	7.4	14	11.7	8	7.8
*Education, n* (%)									
Primary/none		1	0.9	0	0	0	0	0	0
Lower		13	11.2	6	6.3	15	12.5	7	6.9
Middle		22	19.0	17	17.9	20	16.7	17	16.7
Higher		31	26.7	32	33.7	44	36.7	32	31.4
University		49	42.2	40	42.1	41	34.2	46	45.1
*Income, n* (%)									
Low		49	42.2	31	32.6	40	33.3	34	33.3
Middle		20	17.2	21	22.1	34	28.3	26	25.5
High		47	40.5	43	45.3	46	38.3	42	41.2
Medication, *n* (%)		52	44.8	38	40.0	59	49.2	43	42.2
Number of training sessions, mean (*SD*)		5.33	3.95	6.04	4.00	5.17	3.98	6.21	3.93

*ABM* Attentional Bias Modification; *WMT-ED* Working Memory Training with Emotional Distractors

### Training Paradigms

#### Attentional Bias Modification and Control Training

The ABM was a visual search training (de Voogd et al., 2014). Participants had to select the only positive face (an individual depicting a smiling/happy facial expression) as fast as possible in a 4 × 4 matrix of 15 negative faces (an individual depicting an angry, fearful, or sad facial expression; for stimulus selection, see below). The control training was a ‘find-the-flower’ training (Dandeneau et al., 2007) in which participants were presented with a 4 × 4 matrix in which participants had to select the only 5-petaled flower as fast as possible in a matrix of 15 7-petaled flowers. Training sessions started with three practice trials and consisted of 4 blocks of 36 trials in which each trial was repeated until the correct response was given. During the practice trials, feedback was given both after correct and incorrect responses, while during training, feedback was only given after incorrect responses. After each training block, participants received feedback on the number of points that they had earned based on their performance in the previous block.

#### Working Memory Training with Emotional Distractors and Control Training

The emotional chessboard training was used for training WM capacity (De Voogd et al., 2016a). A 4 × 4 matrix was presented of green and blue squares, which lit up in a specific order. Participants had to reproduce the sequence by clicking on the squares in the correct order with the additional instruction to first click on all green squares. The squares lit up in a random order with the constraint that at least one blue square was presented before the last green square. In every sequence, a negative face (angry, fearful, or sad) was presented as distractor on one of the squares that lit up. This square had to be omitted when reproducing the sequence. Feedback was given after each trial and the first training was preceded by 4 practice trials. Additionally, sequence length was dependent on performance, with a minimum of 3 squares which increased or decreased by one square after 2 consecutive correct or incorrect trials respectively. The adaptive WMT-ED consisted of 3 blocks with 12 trials. The control WMT-ED was a non-adaptive version of the same training with a sequence length of 3 squares independent of performance (De Voogd et al., 2016a). As the sequences were shorter in the control training, the number of trials was slightly higher (3 blocks of 15 trials). After each training block, participants received feedback on the number of points that they had earned based on their performance in the previous block.

### Measures

#### Attentional Bias Assessment

AB was assessed with the Emotional Visual Search Task (EVST) (De Voogd et al., 2014). A 4 × 4 matrix of facial expressions was presented in which participants needed to either select a single positive face in a matrix of negative faces (find-positive block) or a negative face in a matrix of positive faces (find-negative block). The task consisted of 2 blocks of 36 trials; one find-positive block and one find-negative block which were counterbalanced across participants. The two blocks were preceded by 6 practice trials, with 3 trials of each type (find-positive or find-negative). During the practice trials, feedback was given both after correct and incorrect responses while during the assessment, feedback was only given after incorrect responses. An AB index was computed by subtracting the average reaction time (RT) for selecting negative faces from the average RT for selecting positive faces (De Voogd et al., 2014). Positive values indicated a bias for negative faces.

#### Working Memory Assessment

WM capacity was assessed with a computerized version (Peeters et al., 2014) of the Self Ordered Pointing Task (SOPT) (Petrides & Milner, 1982). Participants selected concrete or abstract pictures that were presented on a computer screen with the restriction that the same picture or location could not be selected twice. Each time a picture was selected, the pictures were randomly reordered. The SOPT was preceded by 4 practice trials after which the number of pictures per screen increased from 6 to 12 with 8 blocks in total. In case a participant clicked on the same location twice, an extra trial was added to the block. Participants received the number of correct clicks after each incorrect trial and after each block. The total number of correct clicks was used as an index of WM capacity.

#### Face Sets

Face stimuli for the EVST, ABM, and WMT-ED training were drawn from the Umeå University Database of Facial Expressions (Samuelsson et al., 2012) with ages between 17 and 67. Two sets of faces were created with each set containing 36 faces (18 happy faces; 18 negative faces with 6 angry, fearful, and sad expressions). The same set of faces was presented during the pre-training EVST assessment, ABM and WMT-ED training. To investigate whether training effects on the EVST generalized to new faces during mid-, post-, and 3-months follow-up, 50% of the faces were derived from the trained set and 50% were derived from the untrained set. The order of the sets of faces was counterbalanced across participants and a different set of faces was used during the practice trials.

### Questionnaires

Trait anxiety was assessed with trait scale of the Dutch version of the STAI-T (Zelf-Beoordelings Vragenlijst) (Van der Ploeg et al., 1980). The STAI-T is a 20-item self-report questionnaire with a 4-points Likert scale and has good psychometric properties (Van der Ploeg et al., 1980). Cronbach’s alphas were excellent in this study (*α* = 0.91–0.94).

State anxiety was assessed by asking participants to indicate to which degree they experienced anxious feelings at that moment on a visual analog scale from 0 (*Not anxious or tense at all*) to 100 (*Very anxious and tense)*.

After completing all training sessions, or in case participants quit prior to completion, participants received an evaluation questionnaire in which they evaluated the ABM and WM training paradigm separately based on the clarity of instructions and aims of the training, enjoyment, and difficulty. Participants were also asked to indicate which version of the ABM and WMT-ED training they thought they had received (‘real’ or ‘fake’). In case participants quitted prior to completion, they were asked to provide reasons for why they quitted the training.

Demographical questions including age, gender, educational level, income, and use of medication were administered. Participants were asked to indicate their highest educational level, which was categorized as ‘primary/none’ (no education or only primary school), ‘lower’ (only secondary school), ‘middle’ (intermediate vocational education), ‘higher’ (vocational education), and ‘university’ (bachelor degree and up). Additionally, participants were asked to indicate to which of the 3 categories their total gross household income per month belonged to: ‘low’ (less than 2000 euros), ‘middle’ (between 2000 and 3000 euros), and ‘high’ (above 3000).

Based on whether participants indicated if they experienced symptoms of certain disorders in the SCID-I screening questions, corresponding disorder-specific questionnaires were provided. Panic symptoms were assessed with the Panic Disorder Severity Scale—Self Report (PDSS-SR) (Houck et al., 2002). The PDSS-SR is a 7-item questionnaire with a 4-point Likert scale and had good Cronbach’s alpha in this study (*α* = 0.87). Symptoms of specific phobia were assessed with an adapted version of the Fear Questionnaire (FQ) (Marks & Mathews, 1979) in which participants were asked to rate the degree of anxiety and avoidance related to one phobia on a 9-point Likert scale. Obsessive compulsive symptoms were assessed with the Obsessive Compulsive Inventory-Revised (OCI-R) (Foa et al., 2002). The OCI-R is an 18-item questionnaire with a 5-point Likert Scale and had a good Cronbach’s alpha in this study (*α* = 0.88). Posttraumatic stress symptoms were assessed with the Posttraumatic Stress Symptom Scale Self-Report (PSS-SR) (Foa et al., 1993). The PSS-SR is 17-item questionnaire with a 4-point Likert scale and had a good Cronbach’s alpha in this study (*α* = 0.86). Depressive symptoms were assessed with the Beck Depression Inventory–II (BDI-II) (Beck et al., 1996). The BDI-II is a 21-item questionnaire with a 4-point Likert scale and had a good Cronbach’s alpha in this study (*α* = 0.88).

### Procedure

The web-based training was available through an open-access and fully automated website from the Addiction Development and Psychopathology Lab of the University of Amsterdam. The training was referred to as ‘anxiety training’ and was introduced as a training that helps participants ‘to learn new automatic reactions and regain more control about their worries’. Participants were asked to read an information letter and sign a digital informed consent. Then participants received a screening which included the STAI-T, demographic questions, goal formulation, and screening questions of the Structured Clinical Interview for the Diagnostic and Statistical Manual of Mental Disorders Axis I (SCID-I) (First & Gibbon, 2004). To engage and motivate participants before training, participants were asked to fill in advantages and disadvantages of their anxiety symptoms, to formulate which goals they aimed to obtain through participating in the training and to formulate their most important reasons to change and possible barriers to change. Disorder-specific questionnaires were provided based on the SCID-I screening questions. Directly following screening, participants started with the pre-assessment followed by the first training session of active/control ABM combined with active/control WMT-ED.

All training sessions were web-based, and all assessments were self-report questionnaires. Participants were advised to train 3 times a week but were allowed to train on a daily basis. A new session became available 20 h after the previous session was completed. In case participants did not start a new session, reminders were sent after 3, 7, 11, and 21 days, after which participants were warned that the training would not be accessible if they did not start a new session in the next 7 days. Participants could contact research assistants through email in case they had any questions about the training or in case they experienced bugs during training.

Participants completed the assessments at pre-training (T1), mid-training (after 5 training sessions; T2), post-training (T3), at 1-month follow-up (FU1), 2-months follow-up (FU2), and at 3-months follow-up (FU3). Assessments included the STAI-T, EVST, and SOPT (the latter two not at FU1 & FU2). After the last assessment, participants were debriefed and could continue with the active versions of the ABM and WMT-ED. Participants did not receive a financial compensation for participating.

### Data Analyses

Incorrect trials of the EVST (T1: 2.30%; T2: 1.02%; T3: 1.16%; FU3: 1.80%), correct repetitions of incorrect trials, trials deviating more than 2 *SDs* from the individual’s mean or < 200 ms were removed before computing the AB index (De Voogd et al., 2014). Data of participants with an error rate of 3 *SDs* above the mean error rate was excluded (T1: *n* = 6; T2: *n* = 2; T3:* n* = 5; FU3: *n* = 1). To examine training effects on the outcome measures, mixed regression analyses were performed using the *lme4* package version 1.1.13 (Bates et al., 2015) and the *lmerTest* package version 2.0.33 (with the Satterthwaite’s method for approximating degrees of freedom; Kuznetsova et al., 2017; Fai & Cornelius, 1996) using R (R Core Team, 2018). Mixed regression analyses are preferred over a repeated-measures analysis as participants with missing values could be included.

Before performing the mixed regression analyses, a regression model was tested with Time as a continuous predictor (number of months that passed between the assessments) (and condition as categorical predictors). Here, the *n*th degree (depending on the number of assessments) polynomial of Time was added to test which polynomial degree explained the growth curve over time best. The *n*th degree polynomial of Time was chosen based on significance of the polynomial order effects and visual inspection of plots of the data. In each mixed regression model, Participant was entered as the random factor to capture the repeated measures structure and *n*th degree polynomial of Time was entered as a predictor in order to be able to estimate the time curves for individual participants. Likelihood Ratio Tests (LRT) were performed to test whether adding a random regression coefficient for the predictor Time, and a covariance between the random Participant intercept and Time regression coefficient would increase the model fit. As the amount of time between measurements varied within and between participants, an unstructured covariance structure between time points was chosen.

To test whether the active training conditions would outperform the control training conditions, we used the same basic model for all outcome measures in which the main effects and interaction effects of ABM condition, WMT-ED condition, and Time were added. The statistics of the basic models with trait anxiety, AB, and WM capacity as the outcome measures are presented in Appendix 1. The final model was selected by means of a backward elimination procedure and by comparing fit measures of the models (Akaike information criterion and Bayesian information criterion). To test the moderating effects of pre-session state anxiety (averaged over sessions), separate models were tested including the moderator pre-session state anxiety and the interactions with ABM condition, WMT-ED condition, and Time. Exploratively, the moderating roles of baseline AB and WM capacity were tested in separate models with these moderators, their interactions, and time. Furthermore, it was tested whether age, gender, educational level, income, assessment order of the EVST and SOPT, block order of the EVST, and face stimulus set order were significant covariates. Information about log-ins and training sessions were used to determine drop-outs. Drop-out was defined as not completing an assessment or training session. As the high drop-out rate of 47.3% from pre- to mid-assessment affected the model fit, LRTs were performed to test whether adding a covariate (indicating whether or not the mid-assessment was completed) significantly improved model fit. Difference scores between pre- and post-assessment of AB, WM capacity and trait anxiety were computed to investigate whether change in AB or WM capacity significantly correlated with change in trait anxiety.

For all analyses, except for the LRT, Restricted Maximum Likelihood was used. As the same analyses were repeated for 6 outcome measures, a Bonferroni-Holm correction was applied to control for Type I errors. Effects with *p* < 0.05 that did not survive these corrections were defined as marginal. Reference categories were pre-assessment for Time and the control versions of the ABM and WMT-ED. In case of significant training effects, Cohen’s *d* was computed as the difference between the means of two training conditions. The mean was calculated by averaging scores within a certain timeframe (i.e., the average timeframe for completing an assessment ± 1 *SD*), and by dividing this difference by the observed *SD* of that outcome variable at baseline. In case of significant main effects of Time, Cohen’s *d* was computed as the difference between the mean at baseline and the mean of a certain timeframe (mid-assessment, post-assessment, or FU3) divided by the observed *SD* of that outcome variable at baseline.

## Results

### Sample Characteristics with Respect to Baseline Symptoms

The means and standard deviations of the baseline questionnaires are presented per training condition in Table 2. Participants that indicated in the screenings questions of the SCID-I to (maybe) have symptoms of panic disorder (76.0%; *n* = 329) had an average score of 6.20 (*SD* = 5.16) on the PDSS-SR (Houck et al., 2002), which indicates that the severity of symptoms was borderline to slightly ill (Furukawa et al., 2009). Participants that indicated to (maybe) have symptoms of a specific phobia (63.5%; *n* = 275) had an average score of 12.48 (*SD* = 2.94) on the short version of the FQ (Marks & Mathews, 1979). Participants that indicated to (maybe) have symptoms of obsessive–compulsive disorder (79.4%; *n* = 344) had an average score of 14.58 (*SD* = 10.03) on the OCI-R (Foa et al., 2002), which indicates that participants had on average a clinical level of obsessive–compulsive symptoms (Abramowitz & Deacon, 2006). Participants that indicated to (maybe) have symptoms of posttraumatic stress (36.50%; *n* = 158) had an average score of 18.66 (*SD* = 9.14) on the PSS-SR, which indicates that participants had on average a clinical level of posttraumatic stress symptoms (Wohlfarth et al., 2003). All participants also filled in the BDI–II at pre-assessment and had an average score of 19.33 (*SD* = 10.20), which indicates that participants had on average mild depressive symptoms (Beck et al., 1996). Together, the average scores on these baseline questionnaires suggest that the sample of this study was not only characterized by elevated levels of trait anxiety but also borderline to clinical levels of symptoms of other disorders.

**Table 2:** Baseline and outcome measures per training condition at pre-assessment, mid-assessment, post-assessment, and 3-months follow-up

Condition	Outcome measure	T1		T2		T3		FU3	
		*M*	*SD*	*M*	*SD*	*M*	*SD*	*M*	*SD*
ABM & WMT-ED (*n* = 116)									
	STAI-T	56.01	7.05	53.96	8.00	52.58	8.70	50.47	9.32
	EVST	889.95	1217.48	−1124.26	779.60	−1310.88	717.44	−952.00	760.50
	SOPT	59.48	5.36	61.74	5.33	62.46	4.27	63.73	4.34
	PDSS-SR	6.38	5.50	–	–	–	–	–	–
	PTSD-SR	19.20	9.12	–	–	–	–	–	–
	OCI-R	14.20	9.38	–	–	–	–	–	–
	FQ	12.70	2.66	–	–	–	–	–	–
	BDI-II	18.45	10.33	–	–	–	–	–	–
ABM & WMT-ED control (*n* = 95)									
	STAI-T	54.00	7.16	52.82	8.81	51.30	8.30	50.42	8.57
	EVST	728.52	852.50	−1099.32	666.40	−1056.31	647.92	−875.29	841.70
	SOPT	59.10	4.89	58.14	6.12	58.44	5.91	58.87	5.07
	PDSS-SR	4.67	4.27	–	–	–	–	–	–
	PTSD-SR	17.37	8.85	–	–	–	–	–	–
	OCI-R	16.32	11.67	–	–	–	–	–	–
	FQ	12.61	3.15	–	–	–	–	–	–
	BDI-II	19.21	9.01	–	–	–	–	–	–
ABM control & WMT-ED (*n* = 120)									
	STAI-T	55.00	8.17	53.30	8.40	52.14	9.18	46.64	10.83
	EVST	944.00	984.14	859.93	926.87	809.07	1010.89	780.91	706.73
	SOPT	58.14	5.98	59.58	6.09	59.69	6.21	60.82	5.45
	PDSS-SR	6.27	5.20	–	–	–	–	–	–
	PTSD-SR	19.04	9.50	–	–	–	–	–	–
	OCI-R	13.07	9.61	–	–	–	–	–	–
	FQ	11.86	2.80	–	–	–	–	–	–
	BDI-II	21.27	10.26	–	–	–	–	–	–
ABM control & WMT-ED control (*n* = 102)									
	STAI-T	55.15	8.84	52.66	10.16	50.23	11.03	49.59	11.54
	EVST	743.84	1018.30	647.17	1041.66	229.30	1068.92	370.45	913.91
	SOPT	58.22	5.39	61.40	4.77	63.00	4.77	62.84	5.51
	PDSS-SR	7.53	5.28	–	–	–	–	–	–
	PTSD-SR	18.68	9.12	–	–	–	–	–	–
	OCI-R	15.05	9.34	–	–	–	–	–	–
	FQ	12.85	3.09	–	–	–	–	–	–
	BDI-II	18.17	10.81	–	–	–	–	–	–

*ABM* Attentional Bias Modification; *WMT-ED* Working Memory Training with Emotional Distractors; *EVST* Emotional Visual Search Task; *SOPT* Self-Ordered Pointing Task; *STAI-T* State-Trait Anxiety Inventory Trait Scale; *PDSS-SR* Panic Disorder Severity Scale – Self Report; *PTSD-SR* Posttraumatic Stress Disorder Symptom Scale-Self Report; *OCI-R* Obsessive Compulsive Inventory-Revised; *FQ* Fear Questionnaire; *BDI-II* Beck Depression Inventory-II

T1 = pre-training assessment; T2 = mid-training assessment; T3 = post-training assessment; FU3 = 3 months follow-up

### Preliminary Analyses

There were no significant differences between training groups on demographic characteristics (Table 1) nor on outcome measures at baseline (Table 2). An one sample t-test revealed a significant AB for negative information at baseline on the EVST, *t*(426) = 16.65,* p* < 0.001. AB at baseline did not correlate with trait anxiety (*r* = 0.01, *p* = 0.77) nor with WM capacity at baseline (*r* = −0.06,* p* = 0.22). WM capacity did not correlate significantly with trait anxiety (*r* = 0.07, *p* = 0.16). Participants completed on average 5.65 (*SD* = 3.98) of the 10 training sessions. There were on average 1.52 months (*SD* = 0.68) between the completion of the pre- and post-assessment and on average 5.10 months (*SD* = 0.96) between the completion of the pre- and FU3-assessment. Removal of significant covariates in the final models did not change the conclusions drawn by the final model. Covariates of the final models are reported in Table 3.

**Table 3:** Statistics of the final mixed regressions models with trait anxiety, attentional bias, and working memory capacity as the outcome measures

Outcome measures	Parameters	Parameter estimates					
		*B*	*SE*	*P*	*F*	*df*	*p*
STAI-T							
	Time	−0.37	0.05	> 0.001	60.30	1,850.62	> 0.001
	Time^2^	0.08	0.02	> 0.001	20.62	1,788.44	> 0.001
	Time^3^	−0.01	0.00	> 0.001	12.53	1,811.21	> 0.001
	Income	−0.23	0.09	0.01	3.48	2,433.19	0.03
	Block order EVST	−0.18	0.08	0.03	4.95	1,432.10	0.03
EVST							
	ABM condition	−0.13	0.07	0.07	3.31	1,756.95	0.07
	Time	−0.01	0.11	0.94	120.71	1,611.05	< 0.001
	Time^2^	−0.02	0.04	0.57	64.18	1,622.53	< 0.001
	Time^3^	0.00	0.00	0.36	33.13	1,641.15	< 0.001
	ABM condition × Time	−1.77	0.15	< 0.001	139.34	1,733.22	< 0.001
	ABM condition × Time^2^	0.54	0.06	< 0.001	85.88	1,691.79	< 0.001
	ABM condition × Time^3^	−0.04	0.01	< 0.001	54.38	1,685.02	< 0.001
	Age	−0.08	0.03	0.01	6.00	1,409.97	0.01
	Mid-assessment finished	−0.19	0.08	0.01	6.06	1,675.27	0.01
SOPT							
	ABM condition	0.12	0.13	0.34	4.45	1,582.04	0.03
	WMT-ED condition	−0.01	0.12	0.94	0.35	1,582.05	0.55
	Time	1.06	0.14	< 0.001	55.11	1,619.93	< 0.001
	Time^2^	−0.31	0.06	< 0.001	26.20	1,591.65	< 0.001
	Time^3^	0.02	0.01	< 0.001	15.35	1,581.87	< 0.001
	ABM condition × WMT-ED condition	0.12	0.17	0.488	0.48	1,581.91	0.49
	WMT-ED condition × Time	−0.66	0.20	0.001	0.34	1,628.15	0.56
	WMT-ED condition × Time^2^	0.23	0.08	0.005	0.70	1,595.21	0.40
	WMT-ED condition × Time^3^	−0.02	0.01	0.02	0.79	1,583.65	0.37
	ABM condition × Time	−1.01	0.20	< 0.001	9.63	1,628.13	0.002
	ABM condition × Time^2^	0.29	0.08	< 0.001	4.09	1,595.16	0.04
	ABM condition × Time^3^	−0.02	0.01	0.01	1.56	1,583.66	0.21
	ABM condition × WMT-ED condition × Time	1.15	0.28	< 0.001	17.03	1,627.90	< 0.001
	ABM condition × WMT-ED condition × Time^2^	−0.36	0.11	0.001	10.81	1,595.09	0.001
	ABM condition × WMT-ED condition × Time^3^	0.03	0.01	0.01	7.55	1,583.60	0.006
	Age	−0.30	0.04	< 0.001	54.79	1,441.33	< 0.001

*B* = Beta values of the parameter estimates; *SE* = Standard Errors; *ABM* Attentional Bias Modification; *WMT-ED* Working Memory Training with Emotional Distractors; *STAI* State-Trait Anxiety Inventory Trait Scale; *EVST* Emotional Visual Search Task; *SOPT* Self-Ordered Pointing Task. Note that most *p*-values between *p* < 0.01 and* p* < 0.05 are non-significant after Bonferroni-Holm correction. Time^2^ and Time^3^ are referring to the quadratic term of Time (i.e., Time^2^) and cubic term of Time (i.e., Time^3^) in the model

### Training Effects on Trait Anxiety (Primary Outcome Measure)

Our hypothesis that the double active training condition (active ABM & active WMT-ED condition) would outperform the other conditions and that the single training conditions (active ABM & control WMT-ED; control ABM & active WMT-ED) would outperform the double control condition in improving trait anxiety was not confirmed. We observed no significant interaction between ABM condition × WMT-ED condition × Time (*p* = 0.32), nor between ABM condition × Time (*p* = 0.60) or WMT-ED condition × Time (*p* = 0.35). In the final model, we observed a main effect of Time (*p* < 0.001), indicating that trait anxiety decreased from pre-assessment to FU3 independent of condition (Table 3), with a Cohen’s *d* = 0.21 for the decline from pre- to mid-assessment, *d* = 0.35 for the decline to post-assessment, and *d* = 0.76 for the decline to FU3.

### Training Effects on Attentional Bias (Secondary Outcome Measure)

With respect to training effects on AB (cognitive measure; secondary outcome measure), the analyses revealed a significant ABM condition x Time interaction effect *(p* < 0.001; Table 3). Participants who received ABM (ABM & WMT-ED; ABM & WMT-ED control) had a significantly stronger reduction in AB from pre-assessment to FU3 than participants who received ABM control (ABM control & WMT-ED; ABM control & WMT-ED control) (Table 2), with Cohen’s *d* = 1.62 at mid-assessment, *d* = 1.70 at post-assessment, and *d* = 1.53 at FU3. Follow-up analyses with one-way ANOVAs revealed that participants who received ABM had significantly weaker ABs at mid-assessment,* F*(1,207) = 240.26, η^2^_p_ = 0.537, post-mid-assessment*, F*(1,161) = 133.93, η^2^_p_ = 0.454, and at FU3, *F*(1,128) = 104.50, η^2^_p_ = 0.449, compared to participants who received ABM control (all *p*’s < 0.001). The combined training did not outperform the control training conditions in reducing AB, as no significant ABM condition × WMT-ED condition × Time interaction was observed (*p* = 0.05). Exploratively, we did not observe a significant correlation between change in trait anxiety and change in AB from pre- to post-assessment (*r* = 0.002, *p* = 0.98).

### Training Effects on Working Memory Capacity (Secondary Outcome Measure)

With respect to training effects on WM capacity, a significant ABM × WMT-ED × Time interaction (Table 3) was observed. The double active condition (ABM & WMT-ED) outperformed two single training conditions in improving WM capacity, namely the ABM & WMT-ED control condition (*d* = 0.88 at T2, *d* = 0.68 at T3, *d* = 0.70 at FU3) and ABM control & WMT-ED (*d* = 0.51 at T2, *d* = 0.46 at T3, *d* = 0.29 at FU3). The double active condition did not outperform the double control condition (ABM control & WMT-ED control; *d* = 0.16 at T2, *d* = −0.15 at T3, *d* = −0.11 at FU3). Follow-up analyses using planned contrasts in one-way ANOVAs with training condition as a 4-level factor revealed the same pattern: The double active condition significantly outperformed ABM & WMT-ED control and ABM control & WMT-ED at all assessments (ABM & WMT-ED control: T2: *t*(209) = 3.32, η^2^_p_ = 0.050, *p* = 0.001; T3: *t*(163) = 3.28, η^2^_p_ = 0.062, *p* = 0.001; FU3: *t*(128) = 3.69, η^2^_p_ = 0.096, *p* < 0.001; ABM + WMT-ED: T2: *t*(209) = 2.01, η^2^_p_ = 0.019, *p* = 0.046; T3: *t*(163) = 2.25, η^2^_p_ = 0.030, *p* = 0.026; FU3, *t*(128) = 2.24, η^2^_p_ = 0.038, *p* = 0.026). However, the double active condition did not significantly outperform the double control condition (ABM control & WMT-ED control) at all assessments (T2: *t*(209) = 0.32, η^2^_p_ = 0.001, *p* = 0.746; T3: *t*(163) = −0.45, η^2^_p_ = 0.001, *p* = 0.652; FU3, *t*(128) = 0.71, η^2^_p_ = 0.004, *p* = 0.634). The interaction between WMT-ED condition and Time was not significant (Table 3). Finally, the correlation between change in trait anxiety and change in WM capacity from pre- to post-assessment was not significant (*r* = −0.146, *p* = 0.06).

### Moderation of Training Effects

With respect to state anxiety, the average level of state anxiety of all participants before training sessions was 34.00 (*SD* = 21.07). Moderation analyses indicated that, contrary to our expectations, the average level of state anxiety prior to training did not moderate ABM effects on trait anxiety, nor on AB, or WM capacity, see Table 4.

**Table 4:** Moderation of training effects by state anxiety, baseline attentional bias, and baseline working memory capacity

Models with moderators per outcome measure	*F*	*df*	*p*
*STAI*			
ABM condition × Time × State anxiety	0.16	1,861.67	0.69
ABM condition × WMT-ED condition × Time × Baseline EVST	0.05	1,825.28	0.81
ABM condition × WMT-ED condition × Time × Baseline SOPT	0.10	1,827.34	0.75
ABM condition × Time × Baseline EVST	1.67	1,825.28	0.20
ABM condition × Time × Baseline SOPT	0.97	1,827.65	0.32
WMT-ED condition × Time × Baseline EVST	2.46	1,825.77	0.12
WMT-ED condition × Time × Baseline SOPT	3.61	1,827.45	0.06
*EVST*			
ABM condition × Time × State anxiety	0.46	1,743.64	0.50
ABM condition × WMT-ED condition × Time × Baseline SOPT	0.01	1,729.22	0.93
ABM condition × Time × Baseline SOPT	0.48	1,730.69	0.49
WMT-ED condition × Time × Baseline SOPT	0.01	1,730.64	0.92
*SOPT*			
ABM condition × Time × State anxiety	3.43	1,616.18	0.06
ABM condition × WMT-ED condition × Time × Baseline EVST	0.19	1,596.94	0.66
ABM condition × Time × Baseline EVST	0.02	1,597.68	0.89
WMT-ED condition × Time × Baseline EVST	3.59	1,597.67	0.06

*ABM* Attentional Bias Modification; *WMT-ED* Working Memory Training with Emotional Distractors; *STAI * State-Trait Anxiety Inventory Trait Scale; *EVST* Emotional Visual Search Task; *SOPT* Self-Ordered Pointing Task

Exploratively, the moderating role of baseline AB and WM capacity were examined. Results indicated that these baseline cognitive processes did not moderate any training effects on trait anxiety, AB, or WM capacity (Table 4).

### Evaluation of Training Paradigms

The active and control versions of the ABM training and WMT-ED training were evaluated separately (see Table 5). As there were no significant differences on the evaluation questions between the active and control versions of both training paradigms in general (except for the question regarding the difficulty of the training), we described the results of the ABM and WMT-ED training overall.

**Table 5:** Responses to the evaluation questionnaire in the training conditions of the ABM training and the WMT-ED

	ABM	ABM control			WMT-ED	WMT-ED control		
	% (*n*)	% (*n*)	χ^2^(2)	*p*	% (*n*)	% (*n*)	χ^2^(2)	*p*
*The aim of the training was clear before I started*								
Agree	57.89 (44)	62.07 (54)	2.99	0.22	46.67 (35)	42.05 (37)	1.38	0.50
Not agree	22.37 (17)	27.59 (24)			33.33 (25)	42.05 (37)		
Neutral	19.74 (15)	10.34 (9)			20.00 (15)	15.91 (14)		
*The instructions on what to do in the training were clear*								
Agree	96.05 (73)	96.55 (84)	0.70	0.70	89.33 (67)	80.68 (71)	3.76	0.15
Not agree	1.32 (1)	2.30 (2)			4.00 (3)	12.50 (11)		
Neutral	2.63 (2)	1.15 (1)			6.67 (5)	6.82 (6)		
*I enjoyed the training*								
Agree	76.32 (58)	72.41 (63)	0.52	0.77	21.33 (16)	26.14 (23)	0.62	0.73
Not agree	10.53 (8)	10.35 (9)			56.00 (42)	54.54 (48)		
Neutral	13.16 (10)	17.24 (15)			22.67 (17)	19.32 (17)		
*The training task was easy*								
Agree	73.68 (56)	74.71 (65)	0.02	0.99	9.33 (7)	86.36 (76)	99.01	< 0.001
Not agree	11.84 (9)	11.49 (10)			82.66 (62)	9.09 (8)		
Neutral	14.47 (11)	13.79 (12)			8.00 (6)	4.54 (4)		
*Which version of the training do you think you received; the ‘real’ or ‘fake’ one?*								
‘Real’	56.58 (43)	44.83 (39)	1.66^a^	0.20	36.00 (27)	31.82 (28)	0.32^a^	0.57
‘Fake’	43.42 (33)	55.17 (48)			64.00 (48)	68.18 (60)		

*ABM* Attentional Bias Modification, *WMT-ED* Working Memory Training with Emotional Distractors

^a^The degrees of freedom of the χ is 1 instead of 2 for this outcome variable

#### ABM

The majority of the participants enjoyed the ABM training paradigm, thought it was easy, and rated the aim and instructions of the training as clear. Regarding the awareness of training condition, approximately half of the participants thought they received the ‘fake’ version and the other half thought they received the ‘real’ version, and this was not significantly different between the active and control condition, hence participants were blind for their ABM condition.

#### WMT-ED

The majority of the participants indicated that the instructions of the WMT-ED training versions were clear, however, the aim of the WMT-ED training was rated as unclear or neutral. Furthermore, approximately half of the participants did not enjoy the WMT-ED training. The active version of the WMT-ED was rated as significantly more difficult compared to the control version. Regarding the awareness of training condition, the majority of the participants thought that they received the fake version of the WMT-ED training, irrespective of their condition.

### Reasons for Quitting the Training

Of the 72 participants that reported reasons for quitting; 18.0% indicated personal circumstances; 52.78% indicated they were not satisfied with the training; 2.78% indicated that their complaints increased; 12.5% indicated that their complaints decreased; and 2.78% indicated a lack of time.

## Discussion

The aim of this study was to examine the effects of unguided web-based Attention Bias Modification training (ABM) combined with an affective Working Memory training in adults with heightened anxiety. The final sample consisted of 433 adults with elevated levels of trait anxiety as well as borderline to clinical levels of other symptomatology. Approximately 40.6% (*n* = 176) completed all ten training sessions and only 28.9% (*n* = 125) completed FU3. It was hypothesized that active ABM, active WMT, and the combination would outperform the other training combinations in reducing trait anxiety. However, the data did not support this hypothesis, as an overall decline in trait anxiety was observed, irrespective of ABM and WMT training combinations. Concerning effects on cognitive outcome measures, stronger reductions in AB in the ABM training compared to ABM control were observed. With regard to improvements in WM capacity, we observed that ABM combined with WMT (double active) outperformed two other training combinations (ABM + WMT control and WMT + ABM control), but surprisingly not the double control condition. With respect to moderation of training effects; higher levels of state anxiety prior to training did not result in stronger ABM effects, nor did baseline levels of AB or WM capacity moderate effects.

Contrary to expectations, the combination of ABM and affective WMT did not outperform the single training conditions, nor the double control condition in reducing trait anxiety. While theoretical models regarding cognitive vulnerability to anxiety propose that strong AB and low levels of WM capacity jointly determine anxiety (e.g., Ouimet et al., 2009), the current findings suggest that simultaneously modifying both processes does not add anything in anxiety reduction compared to modifying only one of the processes. One possible explanation might be related to the finding that each separate training (either active ABM or active WMT) did not result in stronger reductions in trait anxiety compared to the double control condition. While synergetic effects could occur when combining two types of training, it could be argued that the full potential of combining both training paradigms can only be optimally tested when the individual training-procedures are successful in improving their targeted cognitive processes and anxiety.

With respect to ABM, the finding that ABM training did not result in stronger anxiety reductions than control ABM is in contrast with findings from meta-analyses (Fodor et al., 2020; Linetzky et al., 2015; Price et al., 2016). A recent network meta-analysis (Fodor et al., 2020) showed that when excluding post-traumatic stress trials, ABM significantly reduced anxiety compared to waitlist and control training. There are several potential explanations for this discrepancy. First of all, in almost 90% of the ABM studies a dot probe paradigm has been used (Martinelli et al., 2022), while the current study employed a visual search paradigm. Thus, the type of ABM training might play a role in ABM’s effectiveness in reducing anxiety (see also Vrijsen et al., 2024). Secondly, it has also been shown that delivery setting plays a role as stronger effects have been observed in lab-based or offline studies (Cristea et al., 2015; Fodor et al., 2020; Heeren et al., 2015; Martinelli et al., 2022; Linetzky et al., 2015), while the current study was web-based. Thirdly, previous visual search ABM studies only observed effects on clinician- or parent-rated assessments of symptoms and not on self-report measures (Waters et al., 2013, 2015). This is consistent with meta-analyses indicating larger ABM effects with clinician- versus self-report outcomes (Linetzky et al., 2015; Price et al., 2016). As clinician-rated assessments also include general functioning, these might be more sensitive in capturing changes in daily functioning compared to self-report questionnaires focusing on symptoms only. Or that it takes longer for an individual to recognize change in one-self compared to a trained rater. An interim conclusion might be that web-based, visual search ABM with self-reported anxiety symptoms as an outcome measure might be sub-optimal to achieve and test reductions in anxiety.

A fourth aspect that is relevant to discuss is the impact of ABM on AB. Consistent with previous studies (De Voogd et al., 2016b, 2017; Waters et al., 2015), this study observed a larger decrease in AB after visual search ABM compared to ABM control. However, this decrease in bias was not accompanied by a decrease in trait anxiety. This result is in line with findings from other web-based studies which observed a general decline in anxiety symptoms irrespective of training condition (Boettcher et al., 2012; Carlbring et al., 2012; De Voogd et al., 2016b, 2017). In addition, there was no correlation between change in bias and change in anxiety from pre- to post-training. These results contradict the conclusion of Grafton et al. (2017) who found that if a bias is successfully modified, this is accompanied by a decrease in anxiety. It could be that the observed change in bias could merely reflect a practice or task-specific effect as the ABM training was identical to part of the AB assessment. In line with this idea, some studies suggested that training effects of visual search ABM do not transfer to other assessment tasks (Kruijt et al., 2013; De Voogd et al., 2016b; but see Dandeneau et al., 2007). Together with recent reviews on ABM in anxiety (Mogg et al., 2017), these findings highlight the need for including different bias assessment tasks in order to delineate task-specific versus general changes in AB. Additionally, complementing RT measures with eye-tracking measures might give a more detailed account of changes in bias as RT measures give little insight into the time course before a response and often have low reliability (Waechter & Stolz, 2015).

With respect to affective WMT, this training did not outperform the double control condition in either anxiety reduction or increase in WM capacity. This result is similar to a study using the same WM paradigm with emotional distractors in a general population of adolescents (De Voogd et al., 2016a). However, these findings are in contrast to a recent meta-analysis (Wang et al., 2023) that concluded that WM training had a small, but significant effect on anxiety. There might be several reasons why the affective WMT in the current study might not have affected anxiety. First, given that the working mechanism of WMT is increasing WM capacity, it is not surprising that when the training failed to systematically change WM capacity, no effects on anxiety were observed. Secondly, the performed sub-group analyses in the meta-analysis (Wang et al., 2023) revealed a moderating role of type of anxiety and training setting. While WM training alleviated test anxiety and social anxiety, the effects on trait anxiety (the primary outcome in the current study) were not significant. In addition, offline training resulted in significant effects on anxiety, and effect of web-based training (in the current study) was inconclusive. Thirdly, it should be noted that the current study employed a WM training-variety with emotional distractors, while the assessment did not contain emotional distractors. Hence, the assessment task can be considered a far-transfer task as it contains the same visuospatial aspects but does not contain the emotional context. Perhaps a more near-transfer emotional WM task would have been more sensitive in detecting affective WM capacity changes (see for example Schweizer et al., 2011). Another possible reason why WMT did not outperform its control version is that even though only the active training was adaptive, both training varieties contained an emotional distractor. Thus, in both trainings inhibition of negative emotional stimuli was practiced. Moreover, the WMT was not adaptive with respect to the number of emotional distractors. As inhibition of task-irrelevant information is proposed as a deficit in anxiety (Berggren & Derakshan, 2013), training effects on WM capacity and anxiety might be improved by increasing the number of negative emotional distractors which facilitates inhibition of task-irrelevant stimuli. Lastly, the evaluation questions indicated that the majority of participants rated the active WMT as difficult, not very credible, and with an unclear aim. It might be that the WMT was too challenging and in turn may have limited the training effects by demotivating participants to improve their training performance. As participants were aware that there was a control version, the low credibility might have resulted in making participants less motivated and engaged with the training as they might have thought they had the control version. Engagement with the training can possibly be improved by providing a rationale prior to the training but also provide information on the relevance of the training during the intervention.

Contrary to our expectations, trainings effects were not moderated by pre-session state anxiety, baseline AB, or WM capacity. The absence of moderation effects might be explained by our selected sample of high anxious individuals that might have limited the variability in each of these moderators. Although there was adequate variability in baseline AB and state anxiety, there was little variability in baseline WM capacity. Furthermore, since state anxiety levels were not elicited or manipulated before training (Kuckertz et al., 2014), state anxiety levels were quite low on average which might impede the effects of higher versus lower levels of state anxiety. In order to better evaluate how state anxiety might affect ABM, future studies should consider increasing state anxiety during training (e.g., either by training in an anxious situation or by inducing state anxiety) (e.g., Nuijs et al., 2020) and include assessments of state anxiety *during* training.

This study has several limitations that need to be addressed. First, the drop-out between the pre-assessment and mid-assessment was quite high (41.7–52.6% per condition). While common in web-based studies (De Voogd et al., 2016b, 2017), it might have compromised the interpretability of the final models. The main reason for quitting the training was a low satisfaction with the web-based training which might be best explained by the negative evaluation of the WMT in terms of its enjoyability and rationale. One possible solution could be to offer participants more therapist guidance during the intervention, which is suggested to be an important success factor for web-based interventions (Palmqvist et al., 2007; Spek et al., 2007). Besides the possible benefit of web-based guidance from a therapist, some studies suggest that the user’s experience of cognitive training paradigms can be improved by using virtual reality technology (Otkhmezuri et al., 2019) or more engaging gamified protocols (Notebaert et al., 2018). Second, there was large variability in when participants finished training sessions and completed the following assessments. Hence, participants might have completed post-assessments months after they had finished the training which impedes differentiating between short-and long-term training effects. Although web-based training has advantages as it can be provided at low cost and offers possibilities for wide-scale implementation, it offers little experimental control with respect to the training conditions. Therefore, the findings should not be interpreted as a lack of effectiveness of ABM and affective WM training in more controlled settings such as lab- or clinical contexts (Wiers et al., 2018).

In sum, this study suggests that combining visual search ABM with affective WMT (using a chessboard training paradigm with emotional distractors) in a web-based format is not effective in reducing trait anxiety as we observed general improvements in anxiety over time irrespective of training condition. Consistent with meta-analytic findings, this might be related to the setting of training (web-based versus offline), type of ABM (visual search versus dot probe training), type of anxiety (trait anxiety), or type of anxiety assessment (self-report versus clinician-rated). As web-based interventions that include therapist contact are suggested to be more successful than interventions with no or little therapist contact, testing the effectiveness of cognitive training paradigms in a blended care format might be the way forward.

## Supplementary Information

Below is the link to the electronic supplementary material.

Supplementary file1 (DOCX 42 KB)

## Footnotes

1 Both an anxiety and depression-related training were pre-registered; the current manuscript only focuses on the anxiety-related training. Contrary to the preregistration: alcohol use, depression, anxiety since previous session, and motivation were not analysed given limited power (high drop-out after the pre-assessment). The Liebowitz Social Anxiety Scale, Rosenberg Self-Esteem Scale, and Penn State Worry Questionnaire abbreviated were analyzed in the same manner as the primary outcome measure. The results were similar and presented in Appendix 2. The pre-registered number of completed training sessions was not tested as a moderator as it was too strongly correlated with Time (i.e., participants could only start the next assessment if all training sessions prior to that assessment were completed)
