# Supplementary material for: Combining Unguided Web-Based Attentional Bias Modification and Affective Working Memory Training to Decrease Anxiety: A Randomized Controlled Trial
Source: Cognit Ther Res. 2025 Jan 9;49(4):685–701. doi: 10.1007/s10608-024-10565-y (PMC12287166; doi:10.1007/s10608-024-10565-y)
Supplement: Supplementary file 1 — Supplementary file1 (DOCX 42 KB) [file 10608_2024_10565_MOESM1_ESM.docx]

**Combining Unguided Web-based Attentional Bias Modification and Affective Working Memory Training to decrease Anxiety: A Randomized Controlled Trial**

**Nuijs, M. D. et al.**

**Appendices**

**Appendix 1:** **Statistics of the basic models of the primary outcome measure and cognitive outcome measures.**

**Appendix 2: Results including the statistics of the basic models of social anxiety, self-esteem, and worry (secondary outcome measures).**

**Appendix 1:** **Statistics of the basic models of the primary outcome measure and cognitive outcome measures.**

|  | Outcome measures | | |
| --- | --- | --- | --- |
| Parameters | STAI | EVST | SOPT^a^ |
|  |  |  |  |
| **ABM condition** | *F* = 0.05 | *F* = 3.26 | *F* = 4.45 |
|  | *df =* 1,486.65 | *df* = 1,754.93 | *df* = 1,582.04 |
|  | *P* = .81 | *P* = .07 | *P* = .03 |
| **WMT-ED condition** | *F* = 0.84 | *F* = 2.13 | *F* = 0.35 |
|  | *df* = 1,487.14 | *df* = 1,754.68 | *df* = 1,582.05 |
|  | *P* = .36 | *P* = .14 | *P* = .55 |
| **Time** | *F* = 62.82 | *F* = 120.51 | *F* = 55.11 |
|  | *df* = 1,837.73 | *df* = 1,607.03 | *df* = 1,619.93 |
|  | *P* < .001 | *P* < .001 | *P* < .001 |
| **Time^2^** | *F* = 22.75 | *F* = 63.57 | *F* = 26.20 |
|  | *df* = 1,776.72 | *df* = 1,618.68 | *df* = 1,591.65 |
|  | *P* < .001 | *P* < .001 | *P* < .001 |
| **Time^3^** | *F* = 14.41 | *F* = 32.69 | *F* = 15.35 |
|  | *df* = 1,797.36 | *df* = 1, 636.83 | *df* = 1,581.87 |
|  | *P* < .001 | *P* < .001 | *P* < .001 |
| **ABM condition x Time** | *F* = 0.27 | *F* = 139.73 | *F* = 9.63^**^ |
|  | *df* = 1,839.49 | *df* = 1,726.49 | *df* = 1,628.13 |
|  | *P* = .60 | *P* < .001 | *P* = .002 |
| **ABM condition x Time^2^** | *F* = 0.29 | *F* = 84.79 | *F* = 4.09^*^ |
|  | *df* = 1,777.53 | *df* = 1,685.76 | *df* = 1,595.16 |
|  | *P* = .59 | *P* < .001 | *P* = .04 |
| **ABM condition x Time^3^** | *F* = 0.11 | *F* = 52.98 | *F* = 1.56 |
|  | *df* =1,797.88 | *df* = 1,678.97 | *df* = 1,583.66 |
|  | *P* = .74 | *P* < .001 | *P* = .21 |
| **WMT-ED condition x Time** | *F* = 0.88 | *F* = 0.06 | *F* = 0.34 |
|  | *df* = 1,839.38 | *df* = 1,726.05 | *df* = 1,628.15 |
|  | *P* = .35 | *P* = .81 | *P* = .56 |
| **WMT-ED condition x Time^2^** | *F* = 1.93 | *F* = 0.07 | *F* = 0.70 |
|  | *df* = 1,777.47 | *df =* 1,685.81 | *df* = 1,595.21 |
|  | *P* = .16 | *P* = .79 | *P* = .40 |
| **WMT-ED condition x Time^3^** | *F* = 1.74 | *F* = 0.03 | *F* = 0.79 |
|  | *df* = 1,797.89 | *df* = 1,679.13 | *df* = 1,583.65 |
|  | *P* = .19 | *P* = .86 | *P* = .37 |
| **ABM condition x WMT-ED condition x Time** | *F* = 1.00 | *F* = 3.75 | *F* = 17.03 |
|  | *df* = 1,838.63 | *df* = 1,726.43 | *df* = 1,627.90 |
|  | *P* = .32 | *P* = .05 | *P* < .001 |
| **ABM condition x WMT-ED condition x Time^2^** | *F* = 0.09 | *F* = 1.51 | *F* = 10.81 |
|  | *df* = 1,777.05 | *df* = 1,685.95 | *df* = 1,595.09 |
|  | *P* = .77 | *P* = .22 | *P* = .001 |
| **ABM condition x WMT-ED condition x Time^3^** | *F* = 0.09 | *F* = 0.50 | *F* = 7.55 |
|  | *df* = 1,797.54 | *df* = 1,679.26 | *df* = 1,583.60 |
|  | *P* = .77 | *P* = .48 | *P* = .006 |
| **Income** | *F* = 3.29 | - | - |
|  | *df* = 2,429.91 | - | - |
|  | *P* = .04 | - |  |
| **Block order EVST** | *F* = 5.49 | - | - |
|  | *df* = 1,427.86 | - | - |
|  | *P* = .02 | *-* | *-* |
| **Age** | - | *F* = 6.99 | *F* = 54.79 |
|  | - | *df* = 1,406.09 | *df* = 1,441.33 |
|  | - | *P* = .008 | *P* < .001 |
| **Mid-assessment finished** | - | *F* = 5.18 | - |
|  | - | *df* = 1,672.95 | - |
|  | - | *P* = .02 | - |

ABM = Attentional Bias Modification; WMT-ED = Working Memory Training with Emotional Distractors; STAI = State-Trait Anxiety Inventory; EVST = Emotional Visual Search Task; SOPT = Self-Ordered Pointing Task.

^a^ The basic and final model of the outcome measure SOPT are identical.

Note that most *p*-values between p < .01 and *p* <.05 were non-significant after Bonferroni-Holm correction.

Time^2^ and Time^3^ are referring to the quadratic term of Time (i.e., Time^2^) and cubic term of Time (i.e., Time^3^) in the model.

**Appendix 2: Results including the statistics of the basic models of social anxiety, self-esteem, and worry (secondary outcome measures).**

Social anxiety was assessed with the Liebowitz Social Anxiety Scale (LSAS; Heimberg et al., 1999) and had excellent Cronbach’s alpha’s in the current study (*α* = 0.96-0.97). Self-esteem was assessed with the Dutch version of the Rosenberg Self-Esteem Scale (RSES; Franck et al., 2008) and had good to excellent Cronbach’s alpha’s in the current study (*α* = 0.87-0.91). Pathological worry was assessed with a shortened version of the Dutch Penn State Worry Questionnaire abbreviated (PSWQ-a; Van der Heiden et al., 2009) and had excellent Cronbach’s alpha’s in the current study (*α* = 0.90-0.92). For the mean scores of the RSES, LSAS, and PSWQ per training condition at the pre-assessment, mid-assessment, and post-assessment, and 3-months follow-up, see Table 1.

Our hypothesis that active ABM combined with active WMT-ED would outperform the other training conditions in improving emotional functioning was not confirmed. There were no significant ABM condition x WMT-ED condition x Time interactions or other significant two-way interactions, see Table 2. There were only significant main effects of Time, indicating that social anxiety (*d* = -0.02/0.03/0.28 from pre- to mid-, post-, and FU3-assessment respectively) and pathological worry (*d* = 0.07/0.19/0.40) significantly decreased over time and self-esteem significantly increased over time (Cohen’s *d* = 0.19/0.28/0.48), see Table 2 and 3.

**Table 1.** Means and standard deviations of **s**ocial anxiety, self-esteem and worry per training condition at pre-assessment, mid-assessment, post- assessment, and 3-months follow-up.

| Condition | Outcome measure | T1 | | T2 | | T3 | | FU3 | |
| --- | --- | --- | --- | --- | --- | --- | --- | --- | --- |
|  |  | *M* | *SD* | *M* | *SD* | *M* | *SD* | *M* | *SD* |
|  |  |  |  |  |  |  |  |  |  |
| **ABM & WMT-ED**  **(*n* = 116)** |  |  |  |  |  |  |  |  |  |
|  | LSAS | 50.57 | 25.00 | 51.25 | 22.65 | 49.09 | 25.97 | 43.69 | 25.28 |
|  | PSWQ | 29.04 | 6.24 | 29.31 | 4.76 | 27.26 | 6.21 | 27.13 | 5.25 |
|  | RSES | 24.50 | 5.29 | 24.94 | 4.67 | 26.17 | 4.87 | 26.21 | 5.09 |
| **ABM & WMT-ED control**  **(*n* = 95)** |  |  |  |  |  |  |  |  |  |
|  | LSAS | 50.93 | 26.66 | 56.16 | 23.65 | 52.16 | 19.97 | 51.13 | 21.11 |
|  | PSWQ | 28.76 | 6.07 | 28.37 | 6.68 | 27.23 | 6.07 | 27.68 | 5.98 |
|  | RSES | 24.57 | 4.41 | 25.72 | 4.55 | 26.28 | 4.85 | 26.13 | 5.66 |
| **ABM control & WMT-ED**  **(*n* = 120)** |  |  |  |  |  |  |  |  |  |
|  | LSAS | 51.80 | 27.84 | 52.21 | 27.89 | 48.92 | 25.23 | 44.91 | 31.70 |
|  | PSWQ | 28.48 | 6.31 | 28.00 | 6.15 | 27.27 | 6.32 | 24.27 | 6.63 |
|  | RSES | 24.17 | 4.95 | 24.64 | 5.11 | 26.50 | 5.10 | 28.33 | 5.50 |
| **ABM control & WMT-ED control**  **(*n* =102)** |  |  |  |  |  |  |  |  |  |
|  | LSAS | 52.24 | 30.59 | 48.81 | 30.24 | 41.96 | 31.57 | 42.23 | 31.76 |
|  | PSWQ | 30.38 | 6.03 | 28.69 | 6.13 | 26.65 | 6.59 | 27.75 | 7.08 |
|  | RSES | 24.72 | 5.23 | 25.76 | 6.05 | 27.21 | 6.25 | 26.68 | 5.99 |

ABM = Attentional Bias Modification; WMT-ED = Working Memory Training with Emotional Distractors; LSAS = Liebowitz Social Anxiety Scale; PSWQ = Penn State Worry Questionnaire; RSES = Rosenberg Self-Esteem Scale.

T1 = pre-training assessment; T2 = mid-training assessment; T3 = post-training assessment; FU3 = 3 months follow-up.

**Table 2.** Statistics of the basic models with social anxiety, self-esteem and worry as the secondary outcome measures.

|  | Outcome measures | | |
| --- | --- | --- | --- |
| Parameters | LSAS | RSES | PSWQ |
|  |  |  |  |
| **ABM condition** | *F =* 0.20 | *F =* 0.06 | *F =* 0.32 |
|  | *df* = 1,427.44 | *df* = 1,456.74 | *df* = 1,432.40 |
|  | *P =* .65 | *P =* .80 | *P =* .57 |
| **WMT-ED condition** | *F =* 0.01 | *F =* 0.58 | *F =* 1.08 |
|  | *df* = 1,428.38 | *df* = 1,457.24 | *df* = 1,432.43 |
|  | *P =* .92 | *P =* .44 | *P =* .30 |
| **Time** | *F =* 21.88 | *F =* 36.66 | *F =* 28.76 |
|  | *df* = 1,151.03 | *df* = 1,808.20 | *df* = 1,148.91 |
|  | *P* > .001 | *P* < .001 | *P* < .001 |
| **Time^2^** | *-* | *F* = 12.06 | *-* |
|  | - | *df* = 1,833.07 | - |
|  | - | *P* < .001 | - |
| **ABM condition x Time** | *F =* 1.12 | *F* = 3.25 | *F =* 1.79 |
|  | *df* = 1,150.97 | *df* = 1,809.18 | *df* = 1,148.02 |
|  | *P =* .29 | *P =* .07 | *P =* .18 |
| **ABM condition x Time^2^** | *-* | *F* = 1.96 | *-* |
|  | - | *df* = 1,833.85 | - |
|  | *-* | *P* = .16 | *-* |
| **WMT-ED condition x Time** | *F =* 0.03 | *F* = 0.10 | *F =* 0.11 |
|  | *df* = 1,150.95 | *df* = 1,809.01 | *df* = 1,148.01 |
|  | *P =* .87 | *P* = .75 | *P =* 0.73 |
| **WMT-ED condition x Time^2^** | - | *F* = 0.24 | - |
|  | - | *df* = 1,833.73 | - |
|  | - | *P* = .62 | - |
| **ABM condition x WMT-ED condition x Time** | *F =* 0.26 | *F* = 0.12 | *F =* 0.59 |
|  | *df* = 1,150.98 | *df* = 1,809.14 | *df* = 1,148.03 |
|  | *P =* .61 | *P =* .73 | *P =* .44 |
| **ABM condition x WMT-ED condition x Time^2^** | - | *F* = 0.49 | - |
|  | - | *df* = 1,833.82 | - |
|  | *-* | *P =* .48 | - |
| **Income** | *F* = 10.90 | *F* = 6.64 | - |
|  | *df* = 2,420.94 | *df* = 2,424.92 | - |
|  | *P* > .001 | *P =* .001 | - |
| **Block order EVST** | *F* = 4.49 | - | - |
|  | *df* = 1,420.06 | - | - |
|  | *P =* .03 | - |  |
| **Stimulus set order** | *F* = 5.71 | - | - |
|  | *df* = 1,420.38 | - | - |
|  | *P =* .02 | - | *-* |
| **Age** | - | - | *F =* 8.47 |
|  | - | - | *df* = 1,414.86 |
|  | - | - | *P =* .003 |

ABM = Attentional Bias Modification; WMT-ED = Working Memory Training with Emotional Distractors; EVST = Emotional Visual Search Task; LSAS = Liebowitz Social Anxiety Scale; PSWQ = Penn State Worry Questionnaire; RSES = Rosenberg Self-Esteem Scale.

Note that most *p*-values between p < .01 and *p* <.05 were non-significant after Bonferroni-Holm correction.

Time^2^ and Time^3^ are referring to the quadratic term of Time (i.e., Time^2^) and cubic term of Time (i.e., Time^3^) in the model.

**Table 3.** Statistics of the final models with social anxiety, self-esteem and worry as the secondary outcome measures.

| Outcome measures | Parameters | Parameter estimates | | |  |  |  |
| --- | --- | --- | --- | --- | --- | --- | --- |
|  |  | *B* | *SE* | *p* | *F* | *df* | *p* |
|  |  |  |  |  |  |  |  |
| **LSAS** | Time | -0.05 | 0.01 | < .001 | 22.87 | 1,154.82 | < .001 |
|  | Block order EVST | 0.19 | 0.09 | .04 | 4.35 | 1,432.87 | .04 |
|  | Stimulus set order | -0.22 | 0.09 | .02 | 5.74 | 1,423.91 | .02 |
|  | Income | -0.39 | 0.10 | < .001 | 11.08 | 2,424.11 | < .001 |
| **PSWQ** | Time | -0.08 | 0.01 | < .001 | 30.17 | 1,153.46 | < .001 |
|  | Age | -0.13 | 0.04 | .003 | 8.60 | 1,416.77 | .003 |
| **RSES** | Time | 0.15 | 0.02 | < .001 | 36.45 | 1,815.71 | < .001 |
|  | Time^2^ | -0.01 | 0.00 | < .001 | 11.55 | 1,839.43 | < .001 |
|  | Income | 0.36 | 0.10 | < .001 | 6.72 | 2,428.05 | .001 |

*Note. B* = Beta values of the parameter estimates; *SE* = Standard Errors.

LSAS = Liebowitz Social Anxiety Scale; PSWQ = Penn State Worry Questionnaire; RSES = Rosenberg

Self-Esteem Scale; EVST = Emotional Visual Search Task.

Time^2^ and Time^3^ are referring to the quadratic term of Time (i.e., Time^2^) and cubic term of Time (i.e., Time^3^) in the model.

### **References**

Franck, E., De Raedt, R., Barbez, C., & Rosseel, Y. (2008). Psychometric properties of the

Dutch Rosenberg self-esteem scale. *Psychologica Belgica, 48(1),* 25-35. https://doi.org/10.5334/pb-48-1-25.

Heimberg, R.G., Horner, K.J., Juster, H.R., Safren, S.A., Brown, E.J., Schneier, F.R., &

Liebowitz, M.R. (1999). Psychometric properties of the Liebowitz social anxiety scale. *Psychological Medicine,* *29*(1), 199-212. https://doi.org/10.1017/S0033291798007879

van der Heiden, C., Muris, P., Bos, A.E., van der Molen, H., & Oostra, M. (2009). Normative

data for the Dutch version of the Penn State Worry Questionnaire. *Netherlands Journal of Psychology, 65(2),* 69-75. https://doi.org/10.1007/BF03080129
